# LncRNA RSU1P2 contributes to tumorigenesis by acting as a ceRNA against let-7a in cervical cancer cells

**DOI:** 10.18632/oncotarget.10844

**Published:** 2016-07-26

**Authors:** Qian Liu, Xu Guo, Shengshun Que, Xi Yang, Hongxia Fan, Min Liu, Xin Li, Hua Tang

**Affiliations:** ^1^ Tianjin Life Science Research Center, School of Basic Medical Sciences, Tianjin Medical University, Tianjin, China

**Keywords:** ceRNA, miRNA, Let-7, N-myc, lnc-RNA

## Abstract

Long non-coding RNAs (lncRNAs) can regulate gene expression at different levels and are widely participate in various physiological and pathological processes. Emerging evidences suggests that a number of differentially expressed lncRNAs are involved in tumorigenesis. However, the function and expression regulation of a vast majority of these unique RNAs is little known. Here, we found that the lncRNA Ras suppressor protein 1 pseudogene 2 (RSU1P2) is upregulateded in cervical cancer tissues and has a tumour-promoting role. We revealed that RSU1P2 acts as a competitive endogenous RNA (ceRNA) through regulating the expression of IGF1R, N-myc and EphA4. The mechanism of this regulation is via competition for the shared microRNA let-7a. This competition promotes the malignant phenotype of cervical carcinoma cells. The transcription factor N-myc forms a positive feedback loop with RSU1P2 by in turn activating its expression, thereby enhancing its oncogenic capacity. Hence, cancer-selective targeting of RSU1P2 could have strong benefits.

## INTRODUCTION

The availability of high-throughput technology offers new opportunities to understand genome function through precise and high resolution mapping of the transcriptional genomic landscape. A large number of non-protein coding functional transcripts are encoded by the mammalian genome, as reviewed previously [1, 2]. These non-coding transcripts can generally be divided into two major classes based on their size [3]. Small non-coding RNAs have been relatively well characterized, such as the well-documented microRNAs (miRNAs), which are involved in post-transcriptional regulation of both protein-coding and putatively non-coding genes. In contrast to small non-coding RNAs, very little is known regarding the long non-coding RNA (lncRNA) counterpart of the transcriptome.

By definition, lncRNAs are a class of endogenous non-coding RNAs of more than 200 nucleotides in length that do not code for putative functional proteins [4]. Many identified lncRNAs are transcribed by RNA polymerase II (RNA pol II) and are regarded as the ‘dark matter’ of the transcriptome [5, 6]. However, elegant studies have shown that lncRNAs can regulate gene expression at different levels and widely participate in various physiological processes, including nuclear import, alternative splicing, and epigenetics. These molecules can also act as structural components, as regulators of mRNA decay and even as precursors to small RNAs [7]. Furthermore, accumulating evidence of misregulated lncRNA expression across numerous cancer types suggests that aberrant lncRNA expression may be a crucial component in tumorigenesis [8]. For instance, H19, a 2.3-kb lncRNA, is upregulated in various human cancers, including hepatocellular, bladder and breast carcinomas, suggesting an oncogenic function [9]. Conversely, H19 may also possess tumor suppression properties [10, 11], and epigenetic activation of the miR-200 family contributes to H19-mediated metastasis suppression in hepatocellular carcinoma [12]. It has also been demonstrated that high levels of the lncRNA HOX transcript antisense RNA (HOTAIR) is important in breast cancer development. Many studies have revealed that HOTAIR promotes cancer metastasis and invasion by reprogramming the chromatin state [13, 14]. Moreover, the oncogenic lncRNA metastasis-associated lung adenocarcinoma transcript 1 (MALAT1) has been found to act as a decoy for splicing factors leading to splicing malfunctioning [15].

miRNAs are endogenous ~22-nt RNAs that can play a significant role in most biological processes by targeting mRNAs for cleavage or translational repression [16]. miRNA-binding sequences with partial complementarity to target RNA transcripts are called miRNA response elements (MREs). Importantly, each miRNA has numerous RNA targets, and the vast majority of RNA molecules harbor several MREs and are thus repressed by different miRNAs. On the basis of this logic, Leonardo and colleagues outlined the competitive endogenous RNA (ceRNA) hypothesis, postulating that any RNA transcript that harbors MREs can sequester miRNAs from other targets sharing the same MREs, thereby regulating their expression [17–19]. Therefore, all MRE-containing components of the transcriptome, including mRNAs, transcribed pseudogenes, circRNAs and lncRNA, are capable of regulating each other in this manner [18]. Transcript has potential to be a ceRNA which harboring one or more MREs [20]. The mRNA encoded by ceRNA genes could be involved in distinct biological processes [21]. Such ceRNAs regulate the distribution of miRNA molecules on their targets and thereby impose an additional level of post-transcriptional regulation. Compelling evidence has established that lncRNAs act as “endogenous sponges” that are able to compete for miRNA binding, thereby modulating the de-repression of miRNA targets. Hepatocellular carcinoma upregulated long non-coding RNA (HULC) is highly upregulated in hepatocellular carcinoma and sequesters several miRNAs, including miR-9, leading to de-repression of its target, PPARA, which induces a positive feedback loop involving ACSL1. Thus, HULC's ceRNA activity forms an intricate autoregulatory loop [22].

In the present study, when we reviewed literatures about the regulation of Ras in carcinogenesis, we noticed a lncRNA, Ras suppressor protein 1 pseudogene 2 (RSU1P2). To explore its role in carcinogenesis, we first demonstrated that RSU1P2 was upregulated in cervical cancer tissues compared with adjacent non-tumor tissues and functionied as an oncogene in cervical cancer cells. Furthermore, we revealed that the binding of RSU1P2 to the let-7a miRNA relieved the suppression of the let-7a-targeted genes IGF1R, N-myc and EphA4, which may explain role of RSU1P2 in tumorigenesis of cervical cancer.

## RESULTS

### RSU1P2 is upregulated in human cervical cancer tissues and exerts a tumorigenic function in vivo and in vitro

**Figure 1:**
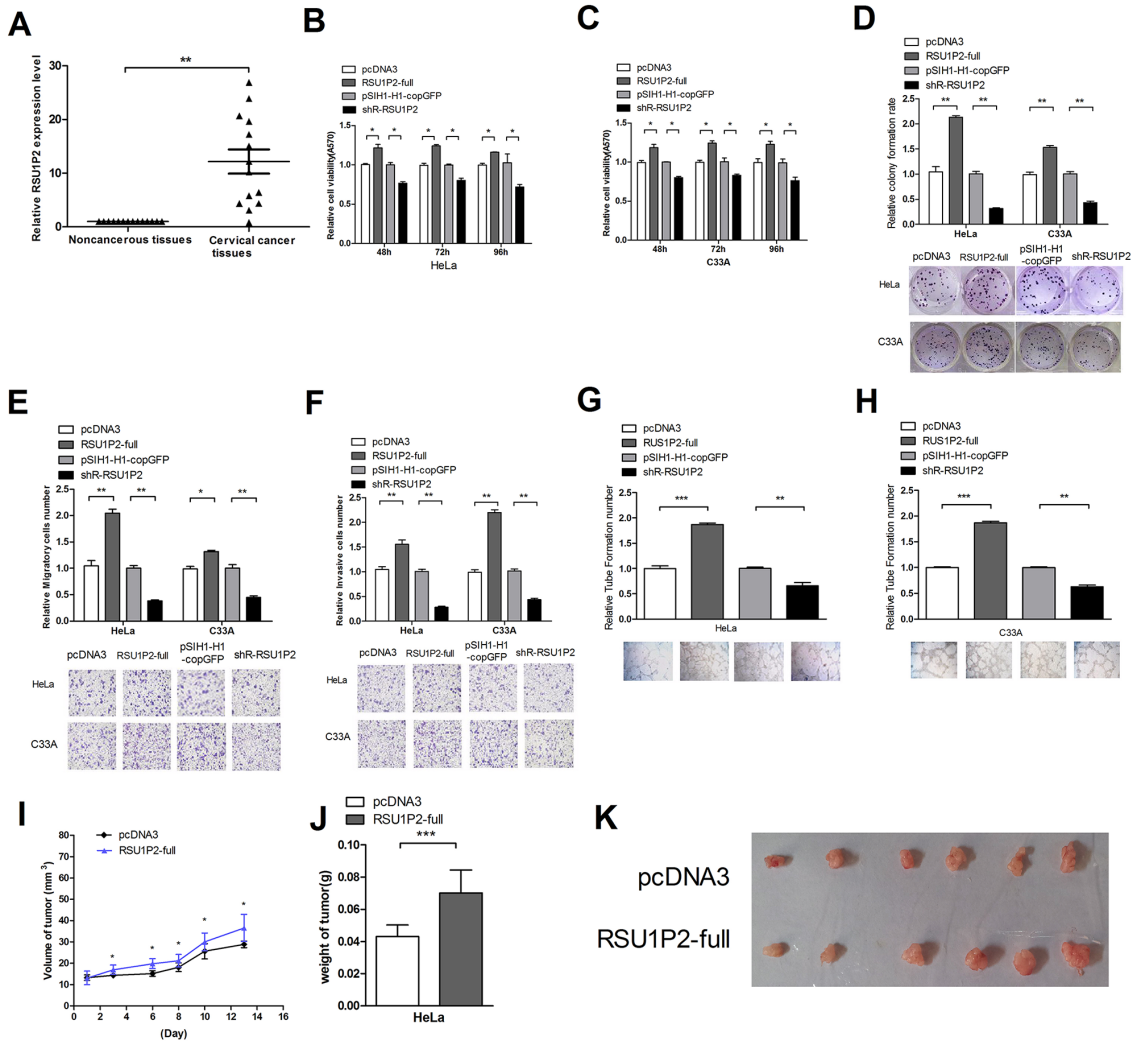
Differential expression of RSU1P2 and RSU1P2 has a growth-promoting role in cervical carcinoma cells **A**.The expression level of RSU1P2 in 14 pairs of cervical carcinoma tissues and matched noncancerous tissues was assayed by qRT-PCR. β-Actin was used as endogenous control. **p<0.01. **B, C**. The cell viability of HeLa and C33A cells in the presence of RSU1P2-full and shR-RSU1P2 was measured at 48 h-96 h post-transfection using an MTT assay. **D**. The cell growth capacity was assessed by colony formation assay. **E**. Transwell migration assay of HeLa and C33A cells after transfection with pcDNA3/RSU1P2-full and shR-RSU1P2. Cells in five random fields of view at 100× magnification were counted and expressed as the average number of cells per field of view. **F**. Transwell invasion assay of HeLa and C33A cells. **G, H**. A vasculogenic mimicry assay was performed to detect the effect of RSU1P2 on angiogenesis in HeLa and C33A cells. *p<0.05, **p<0.01, ***p<0.001. **I, J, K**. The volume and weight of the tumors in the nude mice xenograft experiment showed the effect of full-length RSU1P2 on tumor growth in vivo. Mouse weight and tumor size were measured every 2 days after 7 days of injection. The tumor volume was calculated as follows: length×width^2^×1/2, and the growth curve was drawn (I). The tumor weight was measured when the mouse was sacrificed. The mean weight of the tumors per animal was plotted (J), and all tumors are shown (K). ***p<0.001, n=7. All the mice were sacrificed 20 days after injection.

To investigate the role of RSU1P2 in cervical cancer, we first measured the expression levels of RSU1P2 in 14 pairs of human cervical cancer tissues and adjacent normal tissues using qRT-PCR. The results showed that RSU1P2 expression levels were markedly increased in cervical cancer tissues compared with matched non-tumor tissues (Figure 1A), which prompted us to speculate that RSU1P2 expression may correlate with the malignant behaviors of cervical carcinoma. We also examine the expression level of RSU1P2 in three kinds of cervical cancer cells, Caski, HeLa and C33A, RSU1P2 levels were higher relative to that in Caski cells (Supplementary Figure S1A). In addition, because HeLa is HPV postive cell line and C33A is HPV negative cell line which are representative cervical carcinoma cell lines, we chose HeLa and C33A cell lines to future study.

To evaluate the effects of RSU1P2 on cellular malignancy, we first generated the shRNA vector pSIH1-H1-copGFP/shR-RSU1P2 (shR-RSU1P2) to specifically target RSU1P2 expression and a RSU1P2 expression plasmid, which carried the full-length RSU1P2 complementary DNA (pcDNA3/RSU1P2-full, pRSU1P2-full). After validation of the plasmid's efficiency by qRT-PCR (Supplementary Figure S1B, S1C), we performed functional assays in transfected HeLa and C33A cells with the pcDNA3/RSU1P2-full or shR-RSU1P2 plasmid using a series of experiments. MTT and colony formation assays revealed that significant overexpression of RSU1P2 increased cell viability (Figure 1B and 1C) and colony formation ability (Figure 1D), while RSU1P2 knockdown decreased these phenotypes. In transwell assays, RSU1P2 overexpression increased cell migration (Figure 1E) and invasion (Figure 1F) by approximately 2.1-fold, but sh-RSU1P2 decreased these abilities by approximately 35%. Furthermore, vasculogenic mimicry (VM) analysis showed support for the tumor-promoting role of RSU1P2. The results showed that RSU1P2-full increased the VM of HeLa (Figure 1G) or C33A (Figure 1H) cells by approximately 1.8-fold, but shR-RSU1P2 suppressed it by 40% compared with the controls. These results indicate that RSU1P2 promotes the proliferation, invasion and migration capacity of HeLa and C33A cells.

To further examine the effect of RSU1P2 on tumor growth in vitro, we obtained HeLa pooled clones (HeLa/ RSU1P2-full) that stably expressed higher levels (Supplementary Figure S1D) of RSU1P2 and HeLa/pcDNA3 pooled clones by G418 screening and injected the cells into the flanks of 7 nude mice. At 20 days after injection, the nude mice were sacrificed. The average weight and volume of tumors for the RSU1P2 full-length group was increased compared with the control group (Figure 1I–1K). Together, these results indicate that RSU1P2 may exert tumorigenic functions in vivo and in vitro.

### The let-7 binding region of RSU1P2 facilities tumorigenesis in cervical cancer

**Figure 2:**
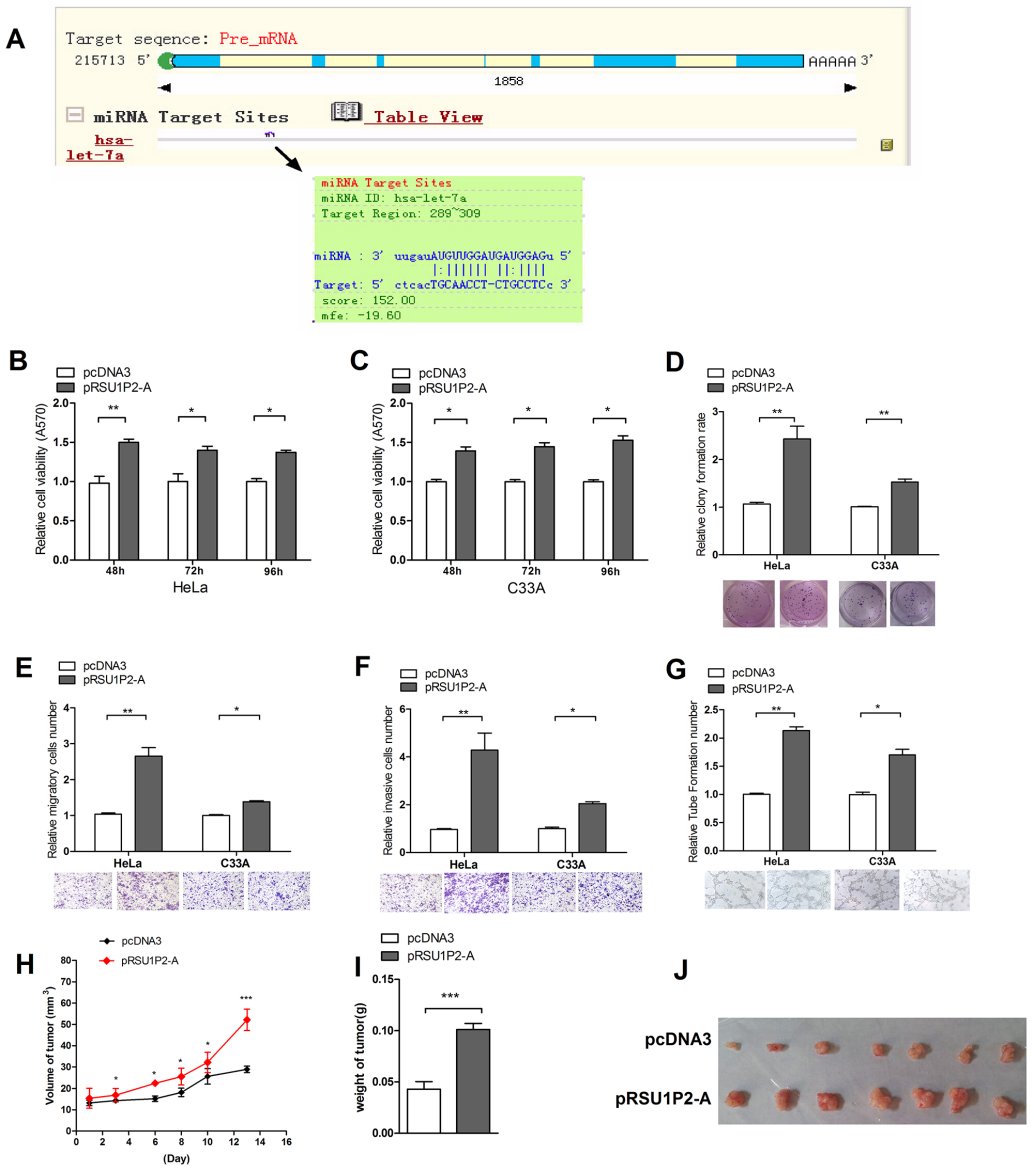
RSU1P2 exerts a tumorigenic function in vivo and in vitro **A**. RegRNA prediction indicated that let-7a could bind to RSU1P2. **B-F**. MTT, colony formation, transwell migration and invasion assays showed that pRSU1P2-A exerts a tumorigenic function in both HeLa and C33A cells. **G**. A vasculogenic mimicry assay was performed to detect the effect of pRSU1P2-A on angiogenesis in HeLa and C33A cells. *p<0.05, **p<0.01, ***p<0.001. **H-J**. The volume and weight of the tumors in nude mice xenograft experiment show the effect of RSU1P2-A on tumor growth in vivo: the tumor growth curve (H), the mean weight (I) and image (J) of each tumor.

Given the pivotal role of RSU1P2 in carcinogenesis, we wished to investigate the mechanism underling its function. There is increasing evidence that lncRNAs regulate corresponding protein-coding mRNAs by acting as a decoy for miRNAs that bind to common sites in the 3′-UTRs. Hence, we hypothesized that RSU1P2 elicits its biological activity through its ability to act as an endogenous decoy for miRNAs. Bioinformatics analysis revealed that the RSU1P2 transcript contains many potential binding sites for various miRNAs (Figure 2A), including the tumor suppressive let-7 family, miR-122 and miR-143, which are downregulated in numerous types of cancer, suggesting that RSU1P2 may act as a ceRNA. According to the known tumor suppressive function of let-7a, we speculated that upregulated RSU1P2 sequesters let-7a to inhibit its tumor-suppressive effects. To avoid other miRNA interference, a 21-bp fragment of RSU1P2 that contains a let-7a binding site was selected for further study to determine whether this region of RSU1P2 mediates, at least in part, the role of RSU1P2 in cervical cancer cells (Figure 2A).

To address this hypothesis, we generated the plasmid pcDNA3/RSU1P2-fragment A (pRSU1P2-A), which contains the sequences of the let-7a binding region of RSU1P2 (Supplementary Figure S2A). Next, we evaluated the effects of RSU1P2-A on malignant phenotypes. As shown in Figure 2B-2F, RSU1P2-A promoted cell viability, proliferation, migration and invasion in HeLa and C33A cells. A VM assay showed that pRSU1P2-A increased the VM formation of HeLa or C33A (Figure 2G) cells compared with control. Pooled cloned HeLa cells, which overexpressed the short RSU1P2 (HeLa/RSU1P2-A), were injected into another 7 nude mice (Supplementary Figure S2B). The average weight and volume of tumors were increased by approximately 110% in the RSU1P2-A group compared with the control vector group (Figure 2H–2J). These results showed that RSU1P2-A exerts a tumorigenic function in vivo. Compared with a striking promotion of growth by full-length RSU1P2 in HeLa cells, a more modest growth promotion and migration/invasion by RSU1P2-WT but not RSU1P2-mutant were observed (Supplementary Figure S3). We also examined the expression levels of RSU1P2-A in the tumors; the results showed that the levels of RSU1P2 in the RSU1P2 group were increased 4-fold (Supplementary Figure S2C) but the levels of let-7a were decreased by approximately 63% (Supplementary Figure S2D) compared with the control group. These data indicate that RSU1P2-A expression promotes the malignancy of cervical cancer cells in vitro and tumor growth in vivo, which is similar to RSU1P2-full. Additionally, the results indicate that RSU1P2-A contains a let-7a binding region, which may mediate the role of RSU1P2.

### RSU1P2 promotes cell cycle progression and EMT and reduces apoptosis

**Figure 3:**
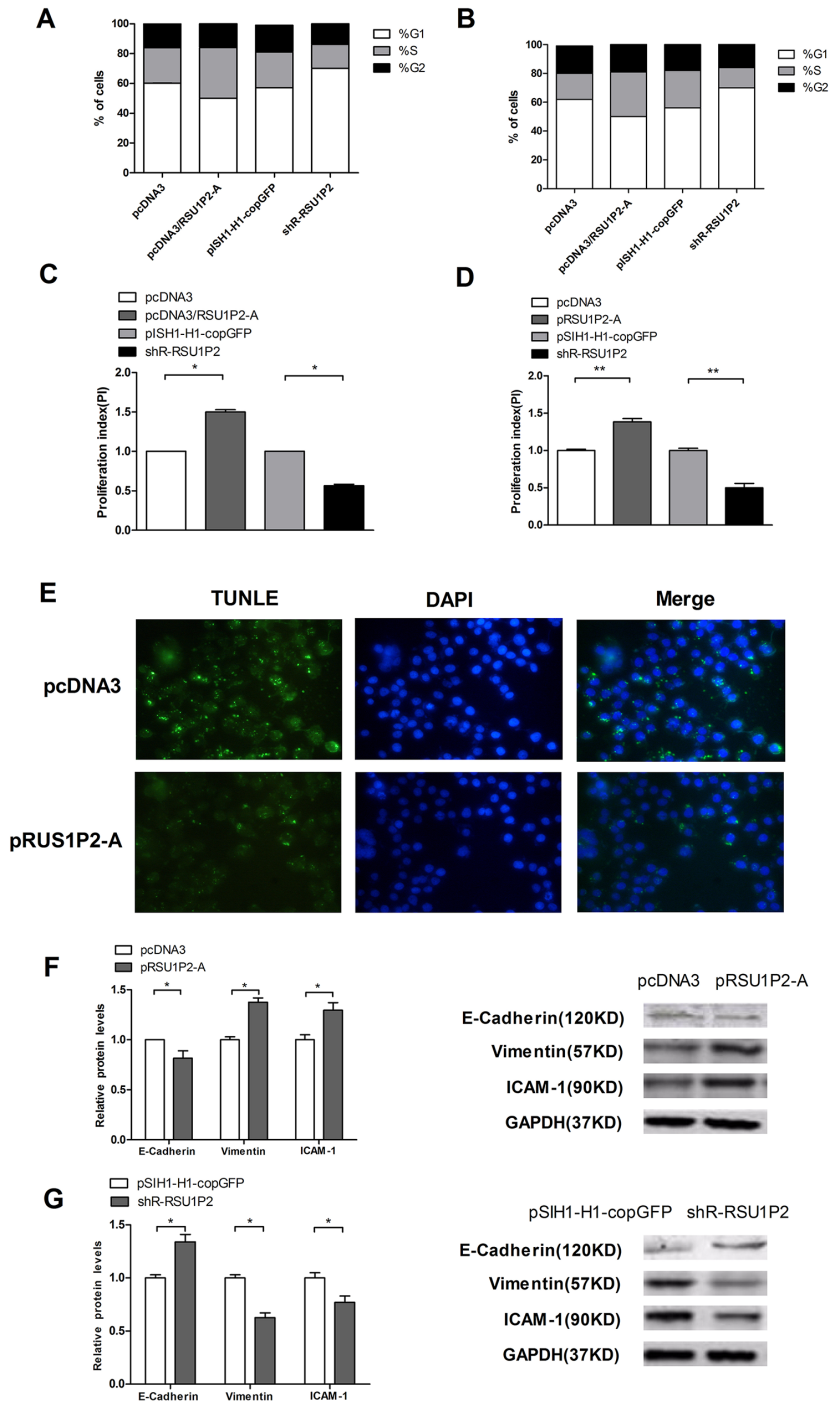
RSU1P2 accelerates cell cycle progression, inhibits cell apoptosis and facilitates the EMT process in vitro **A-D**. Cell cycle progression (by stage) of the transfected cells was analyzed by flow cytometry in HeLa (A) and C33A (B) cells; the proliferation index is shown for HeLa (C) and C33A (D) cells. **E**. TUNEL assay in HeLa cells. The apoptotic bodies are shown by TUNEL (green fluorescence), and the cell nuclei are stained by DAPI (blue fluorescence). **F, G**. The protein levels of E-cadherin, vimentin, and ICAM-1 were examined by immune blotting when RSU1P2 expression was blocked or overexpressed. GAPDH served as a loading control.

Additionally, to explore the mechanism of RSU1P2 in promoting cell growth and proliferation, we investigated the effects of RSU1P2 on the cell cycle and on apoptosis in cervical cancer cells. Due to the similar role of RSU1P2 and RSU1P2-A, we used RSU1P2-A for further study to avoid other miRNA interference. FACS was applied to test the effects of RSU1P2 on the cell cycle. Over-expression of RSU1P2-A resulted in a decrease in the fraction of HeLa cells in G1 phase (from 57.5% to 48.9%) and an increase in S phase fraction (from 22.1% to 30.5%); conversely, knockdown of RSU1P2 in HeLa cells led to an accumulation of cells in G1 phase and decreased entrance into S phase (Figure 3A). The proliferation index of RSU1P2-A-treated cells was higher than that of the negative control, and the index was decreased in HeLa cells transfected with shR-RSU1P2 (Figure 3C). The same phenomenon was observed in C33A cells transfected with pcDNA3/RSU1P2 or shR-RSU1P2 (Figure 3B and 3D). Collectively, these observations strongly suggest that RSU1P2 promotes the G1/S transition in cervical carcinoma cells. Apoptotic cells were visualized by TUNEL assays, and a representative experiment is shown in Figure 3E. The results indicated that cells transfected with pRSU1P2-A exhibited attenuated apoptosis compared with the control group. To determine whether alterations in the EMT is responsible for the promotion of invasion and migration caused by RSU1P2 in cervical carcinoma cells, we performed western blot assays to detect the protein levels of the epithelial marker E-cadherin and the mesenchymal markers vimentin and ICAM-1. RSU1P2-A expression resulted in the downregulation of E-cadherin and the upregulation of vimentin and ICAM-1 (Figure 3F). Conversely, shR-RSU1P2 expression in HeLa cells increased E-cadherin levels, which was accompanied by reduced vimentin and ICAM-1 relative to the control groups (Figure 3G). These results demonstrate that RSU1P2 accelerates the EMT process of HeLa cells. Collectively, these observations provide strong evidence that RSU1P2 promotes tumorigenesis in cervical cancer.

### RSU1P2 is a target of let-7a

**Figure 4:**
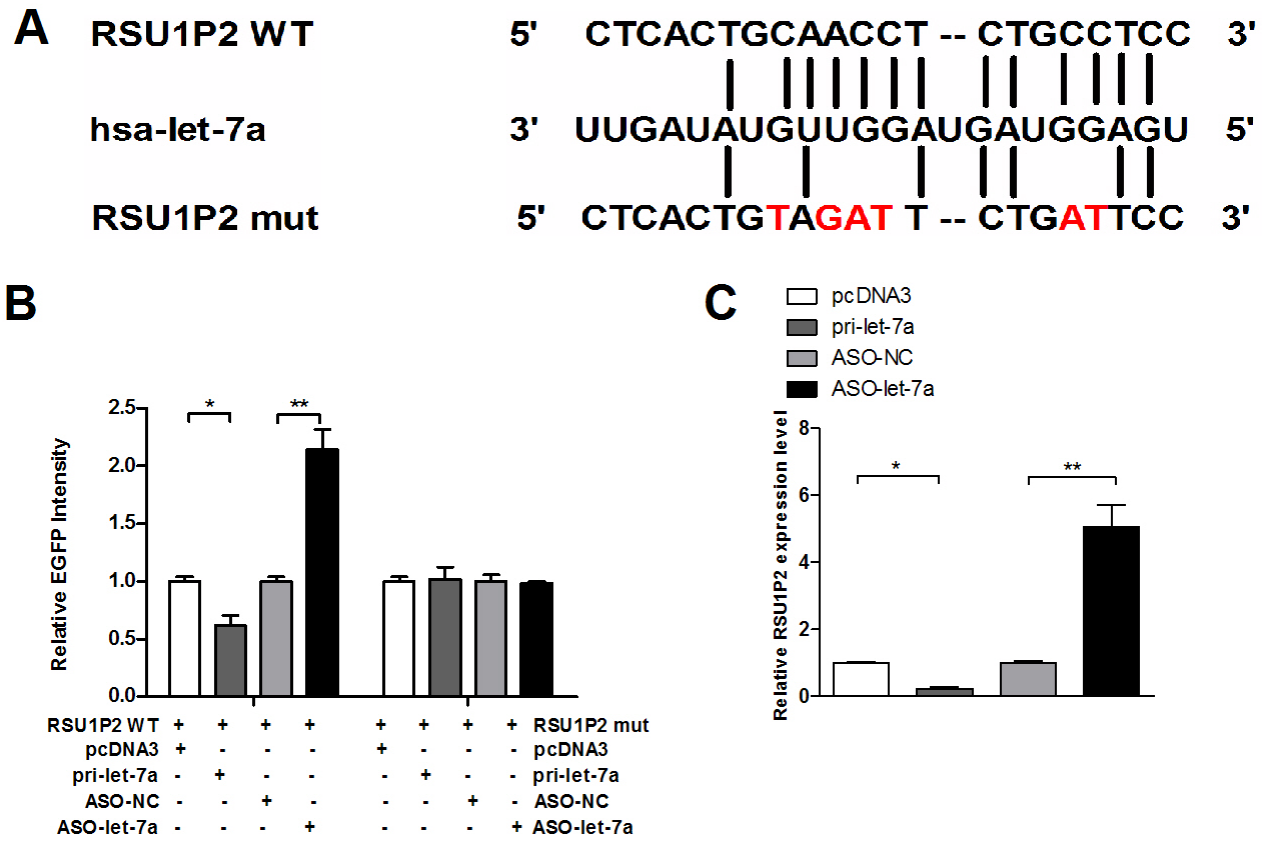
Validation of RSU1P2 as a direct target of let-7a **A**. The sequences of the predicted let-7a binding site and the RSU1P2 segments containing the wild type or mutant binding site are shown. **B**. RSU1P2 (RSU1P2 WT) and mutant derivatives lacking let-7a binding sites were cloned downstream the EGFP coding region. HeLa cells were co-transfected with let-7a or ASO-let-7a, together with the indicated fluorescent reporter vector containing the let-7a binding sites or mutant derivatives of RSU1P2, and an RFP expression vector was used as the loading control. Fluorescent activity was measured 48 h after transfection. **C**. qRT-PCR showed RSU1P2 transcript levels in HeLa cells transfected with let-7a or ASO-let-7a. *p<0.05, **p<0.01.

To confirm that let-7a could bind to RSU1P2, we first constructed a let-7a expression plasmid (pcDNA3/pri-let-7a, pri-let-7a) and validated the efficiency of pri-let-7a and antisense oligonucleotides (ASO-let-7a). Base-pairing complementation revealed that the RSU1P2 sequence contains a putative let-7a binding region (Figure 4A). To validate whether RSU1P2 is a bona fide target of let-7a, the wild type or the mutant fragment with a 6-base mutation in the putative let-7a recognition sequence was cloned downstream of the EGFP reporter gene and transfected in HeLa cells together with pri-let-7a or ASO-let-7a. As shown in Figure 4B, the EGFP intensities were reduced by almost 31% by let-7a expression, whereas ASO-let-7a led to increase EGFP intensities by 2.2-fold compared with the control group when the wild type recognition sequence of RSU1P2 was present. Neither over-expression nor blocking of let-7a had a significant effect on EGFP activity in HeLa cells transfected with the pcDNA3/EGFP-RSU1P2 mutant. Furthermore, qRT-PCR demonstrated that let-7a overexpression dramatically suppressed the endogenous levels of RSU1P2 by 78%, whereas ASO-let-7a induced RSU1P2 by 5-fold in HeLa cells (Figure 4C). These results indicate that let-7a binds RSU1P2 and negatively regulates its expression.

### Identification of RSU1P2 as an ceRNA of let-7a regulating candidate genes

**Figure 5:**
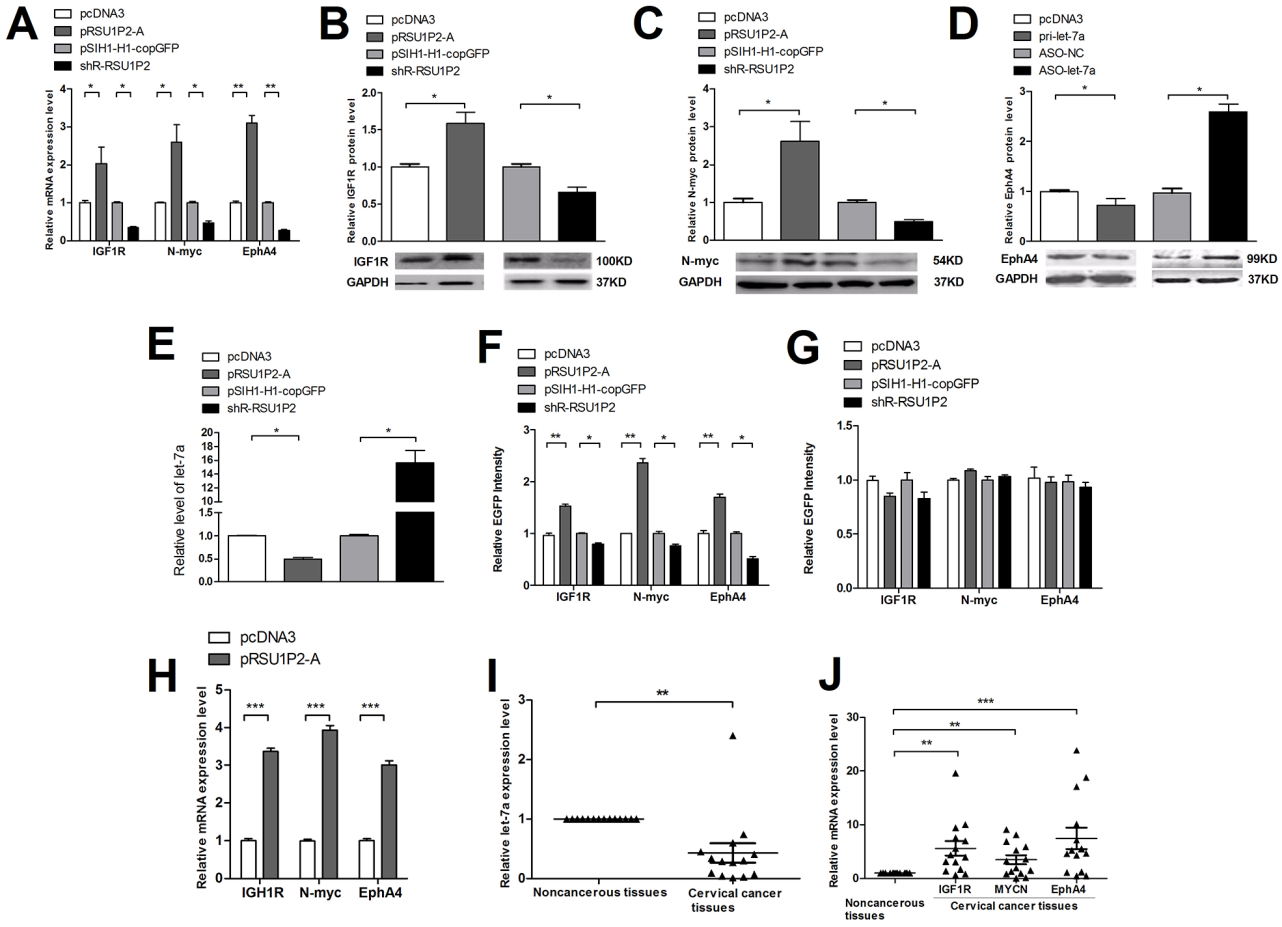
RSU1P2 controls let-7a targets **A-E**. RSU1P2 overexpression de-repressed, but shR-RSU1P2 enhanced, the suppression of both transcript (A) and protein levels (B-D) of the let-7a targets, IGF1R, N-myc and EphA4, and decreased let-7a levels (E). **F, G**. A fluorescent reporter assay indicated the miRNA dependency of RSU1P2-mediated regulation. **H**. The mRNA levels of IGF1R, N-myc and EphA4 in xenograft tumors derived from RSU1P2-A expression in nude mice. **I, J**. The expression levels of let-7a and target gene transcripts in the same 14 pairs of cervical cancer tissues and matched noncancerous tissues as Figure 1A were detected by qRT-PCR. U6 snoRNA and β-Actin were used as endogenous internal controls for normalization. *p<0.05, **p<0.01, ***p<0.001.

We have revealed that RSU1P2 promotes the malignancy of cervical cancer and is downregulated by let-7a. Next, we addressed which let-7a target genes may mediate RSU1P2 function as a let-7a ceRNA. The candidate genes targeted by let-7a were predicted by algorithm programs (TargetScan, miRBase Targets and PicTar). Then, we identified that IGF1R, N-myc and EphA4 were target genes of let-7a using a fluorescent reporter assay, qRT-PCR and western blot analysis (Supplementary Figure S4 and S5). Given that RSU1P2 binds let-7a, abundant RSU1P2 RNA may act as a molecular sponge to sequester let-7a and relieve its repressive function on target genes. Indeed, overexpression of RSU1P2-A in HeLa cells resulted in a de-repression, but shR-RSU1P2 led to inhibition, of both transcript (Figure 5A) and protein (Figure 5B–5D) levels of the let-7a targets IGF1R, N-myc and EphA4. Simultaneously, let-7a levels were inversely related to RSU1P2 levels. As shown in Figure 5E, transfection of pRSU1P2-A resulted in a 50% reduction of let-7a in HeLa cells. Conversely, shR-RSU1P2 led to a marked increase of the let-7a level by ~15-fold. Furthermore, the EGFP reporter vector was generated by inserting the 3′UTR fragment containing the let-7a binding region or a mutant binding region of IGF1R, N-myc and EphA4 to downstream of EGFP (Supplementary Figure S4A). The fluorescent reporter assay was performed by co-transfection of report vector and overexpression or knockdown vector of RSU1P2 in HeLa cells. As shown in Supplementary Figure S4B-S4G, let-7a overexpression significantly suppressed, but ASO-let-7a increased, the EGFP intensities of reporter vectors containing the 3′UTR of IGF1R, N-myc and EphA4. In addition, alteration of let-7a did not affect the EGFP intensities of the reporter vector with a mutant 3′UTR. After validation of IGF1R, N-myc and EphA4 as let-7a targets and let-7a binding of RSU1P2-A, we examined whether RSU1P2-A affects EGFP intensities from reporter vectors of IGF1R, N-myc and EphA4. As shown in Figure 5F, RSU1P2-A overexpression significantly increased the EGFP intensities from the reporter vectors of IGF1R, N-myc and EphA4; conversely, shR-RSU1P2 transcripts significantly reduced these EGFP intensities. However, alterations in let-7a levels had no effect on the EGFP intensities of the 3′UTR mutant vectors of let-7a targets (Figure 5G). qRT-PCR reflect the up-regulated expression of these three target genes in pRSU1P2-A mice compared to the control (Figure 5H). To examine whether the ceRNA activity of RSU1P2 in cervical carcinoma cell lines is relevant to the human disease, we measured let-7a and the RNA levels of its targets in patients samples. qRT-PCR analysis indicated that let-7a expression was downregulated (Figure 5I), but RSU1P2 expression was upregulated (Figure 1A), in cervical cancer tissues compared with adjacent non-tumor tissues. Additionally, IGF1R, N-myc and EphA4 were generally expressed at a high level in 14 pairs of clinical cervical carcinoma tissue samples (Figure 5J). This result indicates that RSU1P2, by binding let-7a, acts as a decoy to abolish the miRNA-repressing activity on IGF1R, N-myc and EphA4 3′UTR.

It has been widely reported that IGF1R mediates cell viability, proliferation and apoptosis in various tumors by signaling pathways, such as IGF and the PI3K pathway, and can be used as a novel targeted therapy [23]. N-myc is a well-known oncogene [24].

**Figure 6:**
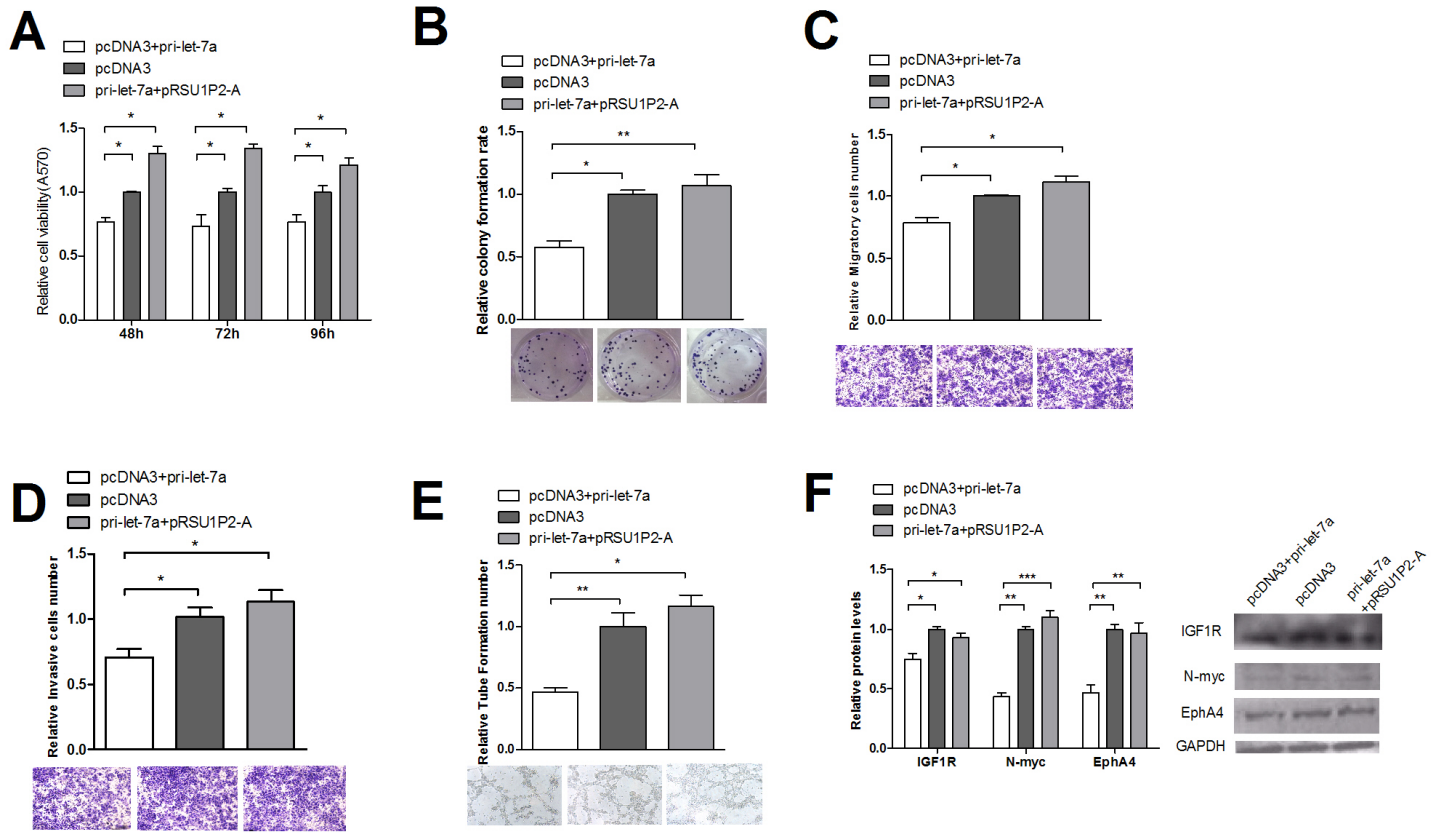
Suppression of let-7a in cervical cancer cells is counteracted by the overexpression of RSU1P2-A RSU1P2-A overexpression rescued the let-7a suppression of cell availability **(A)** colony formation rate **(B)** transwell migration and invasion **(C)** VM **(D)** and protein levels of let-7a targets in HeLa cells **(E)**.

Furthermore, we investigated RSU1P2′s regulation of the endogenous expression of the target genes of let-7a. MTT, colony formation, transwell migration and invasion, VM and western blot assays showed that RSU1P2 could rescue the function of let-7a on malignant phenotypes and target genes (Figure 6A–6F). These data indicate the inverse relationship between RSU1P2 and let-7a levels and the positive relationship between RSU1P2 and let-7a target genes levels in cervical cancer tissue.

To demonstrate that the effect of the RSU1P2 ceRNA is driven by let-7a binding sites, we expressed only the wild type or let-7a-site-mutated segments of RSU1P2 in HeLa cells and examined cell proliferation. Together, these findings provided experimental support for the hypothesis that the RSU1P2 transcript sharing MREs with let-7a targets can regulate the gene expression of the targets by competing for let-7a binding. The existence of a direct relationship between RSU1P2 and let-7a as well as its targets further support our notion that RSU1P2 may perform its oncogenic functions by acting as an “miRNA sponge”.

### N-myc activates RSU1P2 expression to form a positive feedback loop

**Figure 7:**
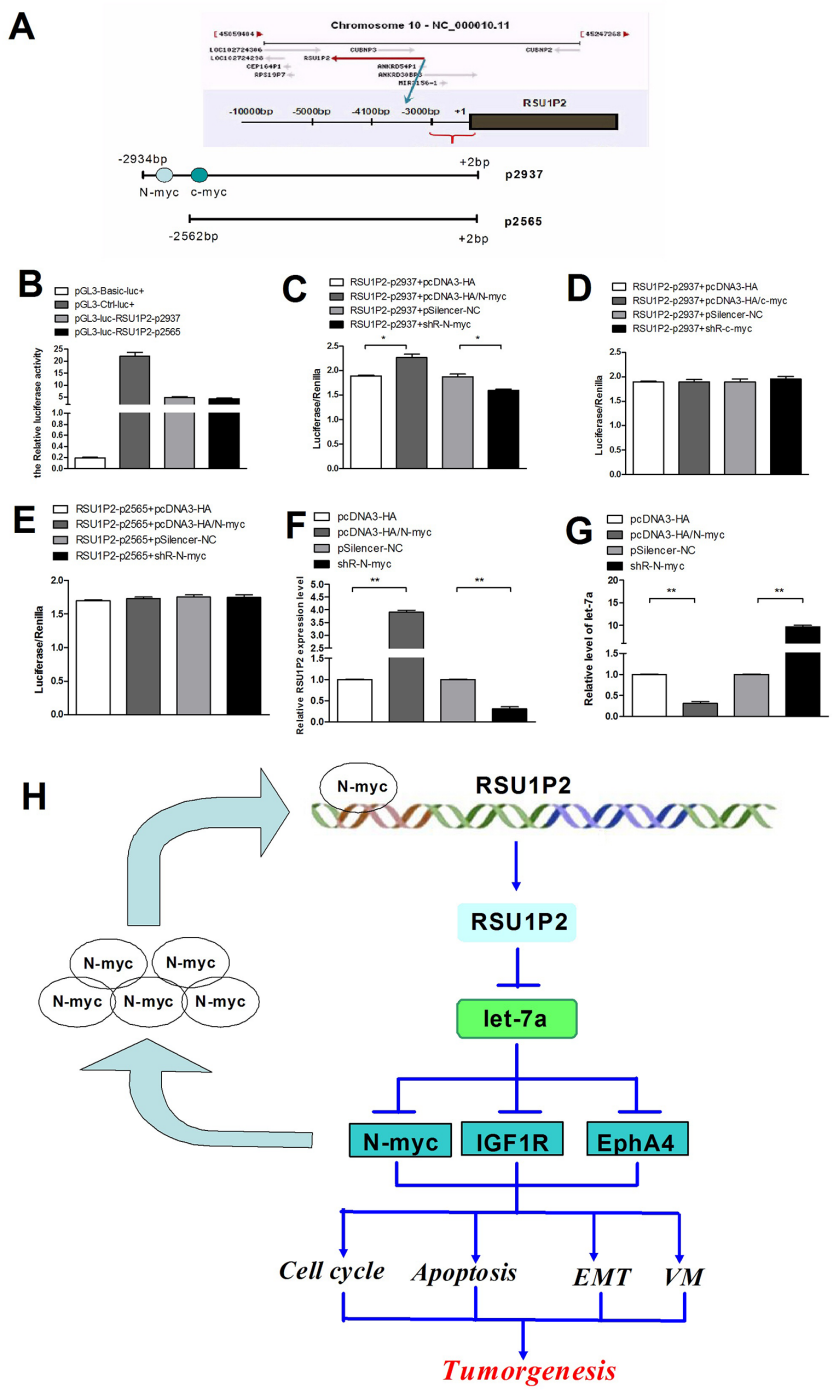
N-myc induces RSU1P2 expression to generate a positive feedback loop of N-myc—RSU1P2—let-7a—N-myc **A**. A diagram of the RSU1P2 promoter fragments. The black area represents RSU1P2. The gray area represents the predicted N-myc and c-myc binding site. **B**. The activities of all the promoter fragments of RSU1P2 on HeLa cells. **C-E**. N-myc (C) but not c-myc (D) enhanced the promoter activity of RSU1P2-2937, and N-myc (E) did not affect the activity of RSU1P2-2937 in HeLa cells. Luciferase reporter assays were performed 24 h after overexpression or knockdown of N-myc and RSU1P2-p2937. The *Renilla* plasmid was co-transfected as a normlization control. **F, G**. qRT-PCR showed that N-myc upregulated the expression of RSU1P2 and downregulated let-7a levels. **H**. The positive feedback loop: N-myc induces RSU1P2 expression, which binds to let-7a to relieve the suppression of N-myc, allowing for an increase in N-myc expression.

To illustrate the mechanism of RSU1P2 upregulation in cervical cancer, we predicted the RSU1P2 promoter by bioinformatics and cloned a 2947-bp fragment upstream from the known RSU1P2 5′ end into pGL3-basic vector (pGL3-basic/ RSU1P2-p2937, RSU1P2-p2937; Figure 7A). Luciferase activity increased almost 5-fold in transfected HeLa cells compared with the control vector (Figure 7B), which indicated that RSU1P2-p2937 possesses the promoter activity of RSU1P2. We predicted that RSU1P2-p2937 contains potential binding sites for N-myc (-2552 bp to -2541 bp) and c-myc (-2453 bp to -2441 bp) by bioinformatics analysis (Figure 7A). To distinguish the effects of N-myc and c-myc, we generated an RSU1P2-p2565 reporter vector that contained the c-myc binding site but not the N-myc binding site. The dual-luciferase reporter assay showed that overexpression of N-myc increased, but shR-N-myc reduced, the luciferase intensity of the RSU1P2-p2937 reporter in HeLa cells (Figure 7C). However, c-myc overexpression or shR-c-myc did not influence RSU1P2-p2937 reporter activity (Figure 7D). In addition, the mutant N-myc binding site in RSU1P2-p2937 abolished the influence of N-myc on luciferase activity (Figure 7E). These results indicate that N-myc and not c-myc activated RSU1P2 promoter activity. Next, we examined whether N-myc could regulate endogenous RSU1P2 expression at the transcriptional level. As shown in Figure 7F-7G, overexpression of N-myc upregulated RSU1P2 expression and downregulated let-7a levels in HeLa cells. Consistently, the knockdown of endogenous N-myc by shR-N-myc significantly reduced the expression of RSU1P2 and increased the level of let-7a. Together, these findings indicate that RSU1P2 is strongly induced by N-myc and binds let-7a to relieve suppression of N-myc (forming a positive feedback loop), which further supports the role of RSU1P2 as an oncogene.

## DISCUSSION

High-throughput studies of mammalian genomes have revealed that the mammalian genome is transcribed into tens of thousands of lncRNAs [1]. Although a small proportion of lncRNAs have been functionally characterized, little is known regarding most lncRNAs. More recently, emerging reports have indicated that lncRNAs are critical players in various cancers, demonstrating potential roles in both oncogenic and tumor suppressive pathways [21, 25–27]. In this study, we reviewed the literature and the lncRNA database and found that RSU1P2 is a pseudogene of Ras suppressor protein 1 (RSU1). RSU1P2 has been shown to have different functions compared with RSU1 in recent reports. RSU1 (~33 kD) suppresses Ras-dependent oncogenic transformation, which promotes cell adhesion and invasion but reduces cell proliferation [28]. Silencing of RSU1 leads to increased cell proliferation and inhibits apoptosis in breast cancer cells [29]. However, the function of RSU1P2 is unclear. Understanding the impact of lncRNAs on physiological and pathophysiological conditions will be of crucial importance to illustrate the function and regulation mechanism of lncRNAs.

The findings presented herein have allowed us to reach a number of important conclusions. First, RSU1P2 is overexpressed in cervical carcinoma tissues and could enhance proliferation, angiogenesis, invasion and migration capacity, promote EMT and the G1/S transformation of HeLa and C33A cells, thus functioning as an oncogene. Second, RSU1P2 sequestered let-7a, effectively perturbing the interaction between let-7a and its targets IGF1R, N-myc and EphA4. Numerous studies have documented that IGF1R and N-myc are target genes of the let-7 family [30,31]. However, this report is the first to show that EphA4 is a target gene of let-7a. IGF1R is dramatically overexpressed in various cancers and possess oncogenic properties through subsequently activating intracellular the PI3K and MAPK pathways, which promote cellular proliferation and metastasis [30, 32, 33]. N-myc, a myc family proto-oncogene, is amplified in 20% of neuroblastoma tumors and is a genetic marker for treatment failure [34]. Notably, it was reported that N-myc could promote cell migration in neuroblastoma cells [35]. EphA4 belongs to the Eph receptor tyrosine kinase family and has been demonstrated to play roles in different types of human cancers. EphA4 promotes cell proliferation and migration through an EphA4-FGFR1 axis in the human glioma U251 cell line [36]. Overexpression of EphA4 gene and reduced EphB2 gene expression correlate with liver metastasis in colorectal cancer [37]. We therefore provide evidence that aberrant regulation of RSU1P2 via miRNA competition by ceRNAs contributes to cervical carcinoma development. Importantly, this observed interaction extended beyond in vitro cell line data to human clinical samples: let-7a levels were decreased in cervical carcinoma tissue samples compared with corresponding adjacent normal tissue, consistent with upregulated RSU1P2 expression. Moreover, bioinformatic analyses identified that RSU1P2 contains multiple binding sites for other miRNAs, such as miR-122, miR-143, and miR-9, leading to the hypothesis that RSU1P2 may regulate the distribution of other miRNA molecules on their targets and thereby combinatorially form an intriguing RNA-RNA crosstalk.

The transcription factor N-myc belongs to the Myc family, in which c-myc, N-myc and L-myc are the best-characterized members. In addition to structural and sequence homologies within the Myc family, the function and biochemical properties of these proteins are closely related [38]. c-myc/N-myc signaling has been demonstrated to activate a core set of miRNAs directly binding to the E-box, providing further support for the overlapping functions of N-myc and c-myc in tumorigenesis [39]. We predicted the promoter region of RSU1P2 contain potential binding sites of N-myc and c-myc, but didn't observe that c-myc significantly affected the reporter activity as shown in Figure 7D, which suggest that the predicted c-myc bind site may be weak activity. Recently, a rapidly growing number of subsequent studies confirmed that N-myc affects the expression of ncRNAs, including miRNAs (mir-17-92 cluster, miR-9 and miR-421) and lncRNAs [40]. Moreover, let-7, miR-34a and miR-101 have been shown to be strong negative regulators of N-myc expression [41,42]. In our research, we showed that let-7a could target the 3′-UTR of N-myc and inhibit its mRNA and protein production. Bioinformatics analysis predicted that N-myc could bind the promoter region of RSU1P2. Combined with qRT-PCR, we verified that overexpression of N-myc could upregulate RSU1P2 expression and downregulate the level of let-7a. These findings indicate the existence of a positive feedback loop, where proto-oncogene N-myc induces RSU1P2 expression at the transcriptional level and then represses the inhibition of let-7a on its target N-myc, whereas let-7 inhibits N-myc protein production (Figure 7F). In addition, TCGA data showed that RSU1P2 is high-expression in breast cancer, bladder cancer and prostate cancer (Supplementary Figure S6), it needs to be unraveled whether that RSU1P2 exerts the similar role in these cancer cells.

In summary, the results from our study show that RSU1P2 contributes to carcinogenesis through regulating the distribution of let-7a on its targets. Understanding the effect of RSU1P2 on the cellular malignancy will help elucidate the pathogenesis of aggressive cervical carcinoma and provide a basis for novel targeted therapies for cervical carcinoma treatment.

## MATERIALS AND METHODS

### Clinical specimens and RNA isolation

Paired human cervical carcinoma tissue samples and adjacent normal cervical tissue samples from 14 cervical cancer patients were obtained from the Tumor Bank Facility of Tianjin Medical University Cancer Institute and Hospital and the National Foundation of Cancer Research, with patient informed consent, which was approved by the ethics committee. The categorization of the clinical samples was confirmed by pathological analysis. A detailed procedures is available elsewhere [43].

### qRT-PCR analysis

A detailed procedures is available elsewhere [43]. Quantitative RT–PCR was performed to detect the relative transcript levels of RSU1P2, let-7a and its target genes. The paired primers are described in Supplementary Table S1. qRT-PCR analysis was performed in triplicate with the SYBR Premix Ex Taq™ kit (TaKaRa) according to the manufacturer's instructions.

### Cell culture and transfection

The human cervical cancer-derived cell lines, HeLa and C33A, were cultured in RPMI1640 (Invitrogen) supplemented with 10% fetal bovine serum (FBS), 100 μg/ml streptomycin, and 100 IU/ml penicillin and maintained at 37°C in a humidified atmosphere with 5% CO_2_. Cell transfection was performed with Lipofectamine 2000 Reagent (Invitrogen), following the manufacturer's protocol.

### miRNA target prediction

Using the most frequently used prediction algorithms of RegRNA, TargetScan and PicTar, we identified the putative downstream mRNA target of let-7a.

### Western blot analysis

A detailed procedures is available in supplemental information. The membranes were then probed with specific primary antibodies. The rabbit anti-human GAPDH, MYCN, IGF1R, EphA4 primary antibodies were from Saierbio (Tianjin, China), and rabbit anti-human bak was from Santa Cruz Biotechnology (CA, USA). The resulting Western blot bands were quantified using the LabWorks™ 4.0 software.

### Detection of cell viability and proliferative capacity

The 3-(4,5-dimethylthiazol-2-yl)-2,5-diphenyl-tetrazolium bromide (MTT) assay and cell proliferation assay was performed to evaluate the viability and proliferative capacity of cervical carcinoma cell lines. For the MTT assay, 8000 C33A cells or 5000 HeLa cells were seeded into 96-well plates 20~24 h after transfection. For the colony formation assay, 300 C33A cells or 250 HeLa cells were plated into 12-well plates as above. A detailed procedures is available elsewhere [43]. All experiments were performed more than three times.

### Cell cycle by flow cytometry

Transfected C33A or HeLa cells were plated in 6-well plates in duplicate for 24 h in complete culture solution. One group of cells was deprived of serum for 24 h before harvesting, while another group of cells was returned to complete medium for another 24 h before harvesting. The cells were gathered by centrifugation, fixed in 95% (V/V) ethanol, and stored at -20°C overnight. After washing with phosphate-buffered saline (PBS), the cells were resuspended in propidium iodide (PI) staining buffer (PBS, 0.1% Triton X-100, 50 μg/ml PI, 0.1 mg/ml DNase-free RNase, and 0.1% trisodium citrate) for 30 min on ice. The DNA content was analyzed with FACS Calibur flow cytometer (BD Biosciences) and Cell Quest software (BD Biosciences).

### TUNEL assay

HeLa cells were transfected with pcDNA3/RSU1P2 or the control. At 48 h after transfection, the cells were washed by PBS and fixed with 4% paraformaldehyde in PBS at room temperature for 30 min. After incubation in PBS for 5 min, the cells were permeabilized for 2 min with 0.1% (v/v) Triton X-100 on ice. The samples were washed twice with PBS for 5 min each, incubated for 1 h in mixed solution (enzyme solution:label solution = 1:9, Roche) and protected from light. Before staining with DAPI (4, 6-diamidino-2-phenylindole, 300 nmol/L from Invitrogen), the cells were washed in PBS three times, 5 min each. After the final wash, the samples were visualized with an imaging system (NIS Elements F 2.20 imaging software, Nikon, Tokyo, Japan).

### EGFP reporter assay

HeLa cells were transfected with pcDNA3/pri-let-7a, control vector, let-7a antisense oligonucleotide (ASO), control ASO (ASO sequences are listed in Supplementary Table S1), and the reporter plasmids described above in 48-well plates. An expression vector expressing red fluorescent protein (RFP), pDsRedz-N1 (Clontech), was used for normalization. The cells were lysed with RIPA lysis buffer 48 h later, and the protein was harvested. EGFP expression and RFP expression were detected using a fluorescence spectrophotometer F-4500 (Hitachi). All assays were performed more than three times.

### Cell migration and invasion assay

Cell migration assays were performed in 24-well transwell chambers (Corning, Cambridge, MA, USA). A detailed procedures is available in supplemental information.

### Plasmid construction

We inserted a DNA fragment containing the pri-let-7a gene DNA into pcDNA3 vector between the BamHI and EcoRI restriction sites, yielding the plasmid pcDNA3/pri-let-7a (pri-let-7a).

We performed an annealing reaction using two single strands. The wild type or mutant form of the target gene mRNA 3′ UTR was inserted into the downstream region of the pcDNA3/EGFP vector between BamHI and EcoRI sites.

We obtained the plasmid, pUC57-RSU1P2 (GENEWIZ), which contained full-length RSU1P2, then reconstructed the fragment into pcDNA3 vector between BamHI and EcoRI sites. All primers used are listed in Supplementary Table S1, and all constructs were confirmed by DNA sequencing.

### Tumor xenograft model in nude mice

HeLa cells were transfected with pcDNA3/RSU1P2 and the control vector. A total of 3×10^6^ transfected cells were suspended in 100 μl of serum-free RPMI1640 culture medium and subcutaneously injected into 6 week-old female nude mice in the oxter. Mouse weights and tumor sizes were measured every 2 days after 7 days of injection. The tumor volume was calculated as follows: length×width^2^×1/2. All mice were sacrificed 20 days after injection. The tumors were isolated from the mice and stored at −80°C. All studies were performed under the American Association for the Accreditation of Laboratory Animal Care guidelines for the humane treatment of animals and adhered to national and international standards.

### Statistical analyses

The data are expressed as the mean±S.D. from at least three separate experiments. The differences between groups were analyzed using double-sided Student's test, and significance was determined by a *p* value of less than 0.05.

## SUPPLEMENTARY MATERIALS FIGURES AND TABLES




