# Supplementary material for: LncRNA RSU1P2 contributes to tumorigenesis by acting as a ceRNA against let-7a in cervical cancer cells
Source: Oncotarget. 2016 Jul 26;8(27):43768–81. doi: 10.18632/oncotarget.10844 (PMC5546439; doi:10.18632/oncotarget.10844)
Supplement: Supplementary file 1 [file oncotarget-08-43768-s001.pdf]

RSU1P2 contributes to tumorigenesis by acting as a ceRNA against let-7a in cervical cancer cells

EXPERIMENTAL PROCEDURES

Cell migration and migration assay

A total of 5×10^4 HeLa cells or 8×10^4 C33A cells in 200 μL of serum-free RPMI1640 were trypsinized, washed, resuspended in serum-free RPMI1640 and seeded in the top portion of the chamber. Another 700 μL of RPMI1640 containing 20% FBS was added to the lower portion of the chamber as a chemoattractant. The chambers were incubated at 37°C in 5% CO_2 for 48 h (HeLa and C33A cells), washed with PBS, and fixed in fixing solution with 25% methanol and 75% acetic acid. The fixed cells were stained with crystal violet and imaged, and the number of invasive cells was counted. Five random fields were analyzed for each chamber. The assays were conducted in three independent experiments.

In the cell invasion assay, pre-chilled serum-free RPMI1640 was mixed with Matrigel (1:4; BD Biosciences, Franklin Lakes, USA). The upper compartments of the chambers were filled with 40 μl of the mixture, and the Matrigel was allowed to solidify at 37°C for 1 h. A total of 8×10^4 HeLa cells or 12×10^4 C33A cells in 200 μL of serum-free RPMI1640 was seeded in the top portion of the chamber. The remaining protocol was the same as that of the cell migration assay.

Western blot analysis

Transfected cells were washed in chilled PBS and lysed directly in the wells by incubating on ice for 30 min with radio immunoprecipitation assay lysis buffer (1 mM $MgCl_2$, 10 mM Tris-HCl, pH 7.4, 1% Triton X-100, 0.1% SDS, 1% Nonidet P-40). An equal amount of protein was separated on 10% SDS denaturing polyacrylamide gel and transferred to nitrocellulose membranes.

SUPPLEMENTARY FIGURES AND TABLE

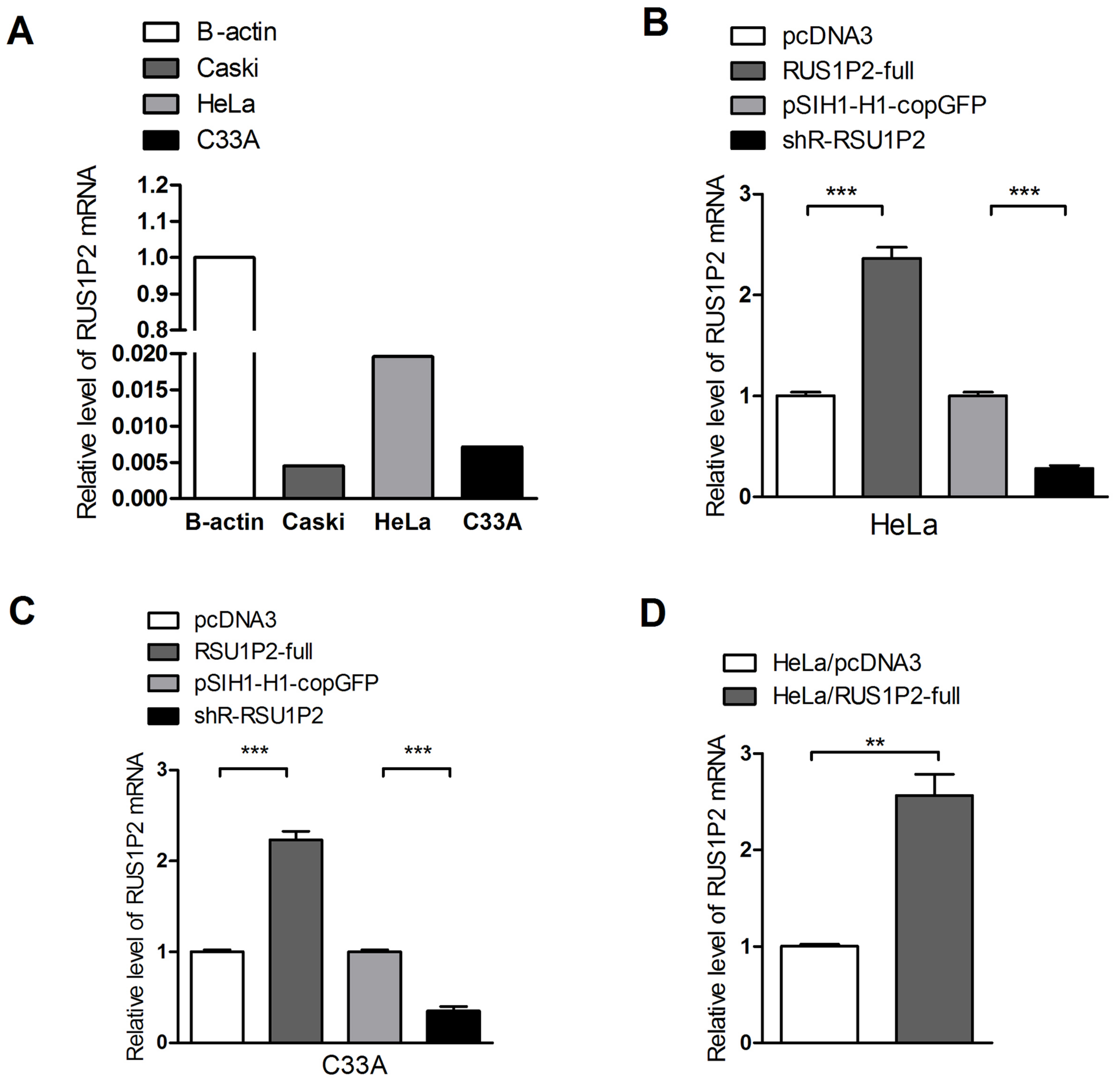

Supplementary Figure S1: The relative level of RSU1P2 in cervical cancer cells and the validity of plasmids for overexpression and knockdown RSU1P2 and RSU1P2-full pooled clone cell line. **A.** The relative level of RSU1P2 in cervical cancer cells. **B.** In HeLa cells. **C.** In C33A cells. **D.** In the RSU1P2-full overexpression pooled clone cell line.

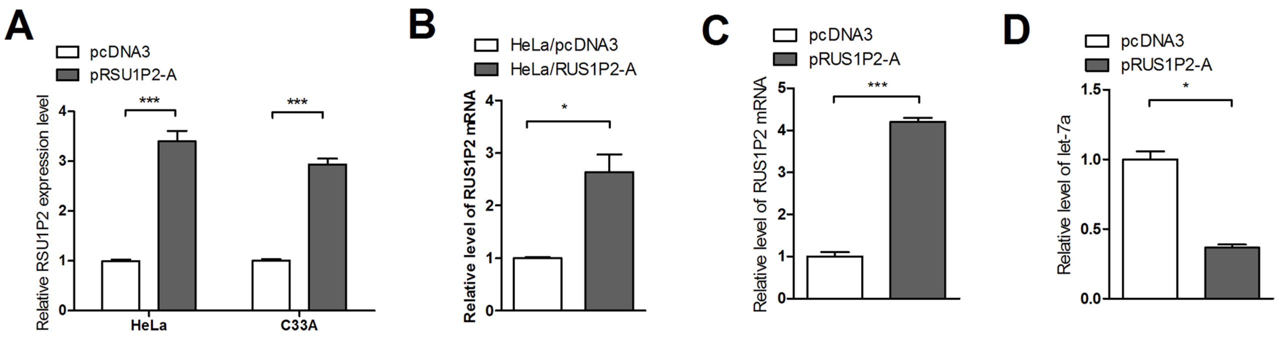

Supplementary Figure S2: qRT-PCR examination of RSU1P2 and let-7a levels in various cells. A, B. The validity of the pcDNA3/RSU1P2-A plasmid and HeLa/RSU1P2-A pooled clone cell line. **C, D.** The relative levels of RSU1P2 and let-7a in the mouse tumors transfected with pcDNA3/RSU1P2 compared with the control group.

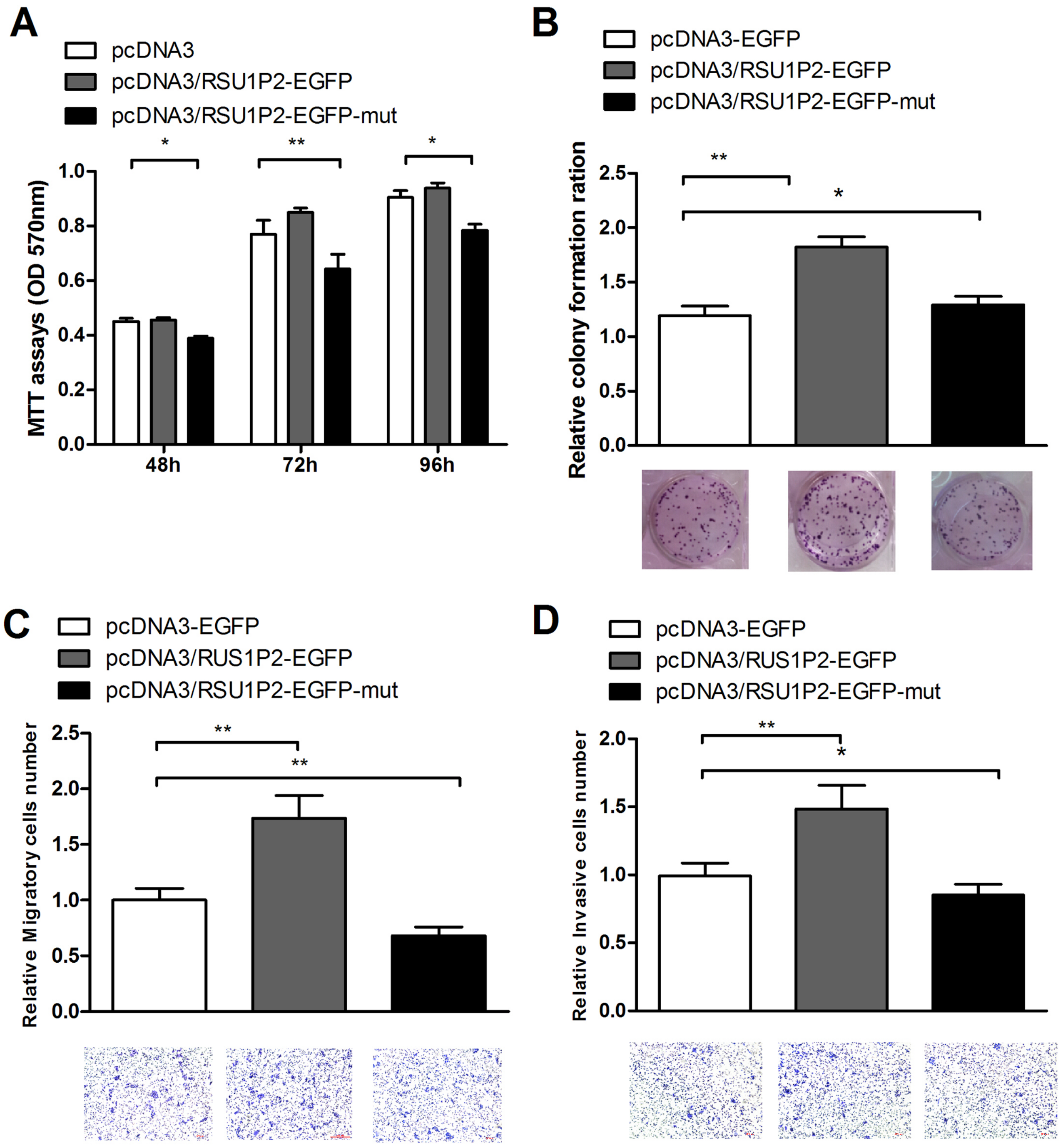

Supplementary Figure S3: Let-7a-induced phenotypes could be rescued by RUS1P2 MTT A. colony formation **B.** cell migration **C.** and invasion assay **D.** showed that the promotion of growth induced by RSU1P2 could be abrogeted when RSU1P2-mutant of let-7a binding site was transfected.

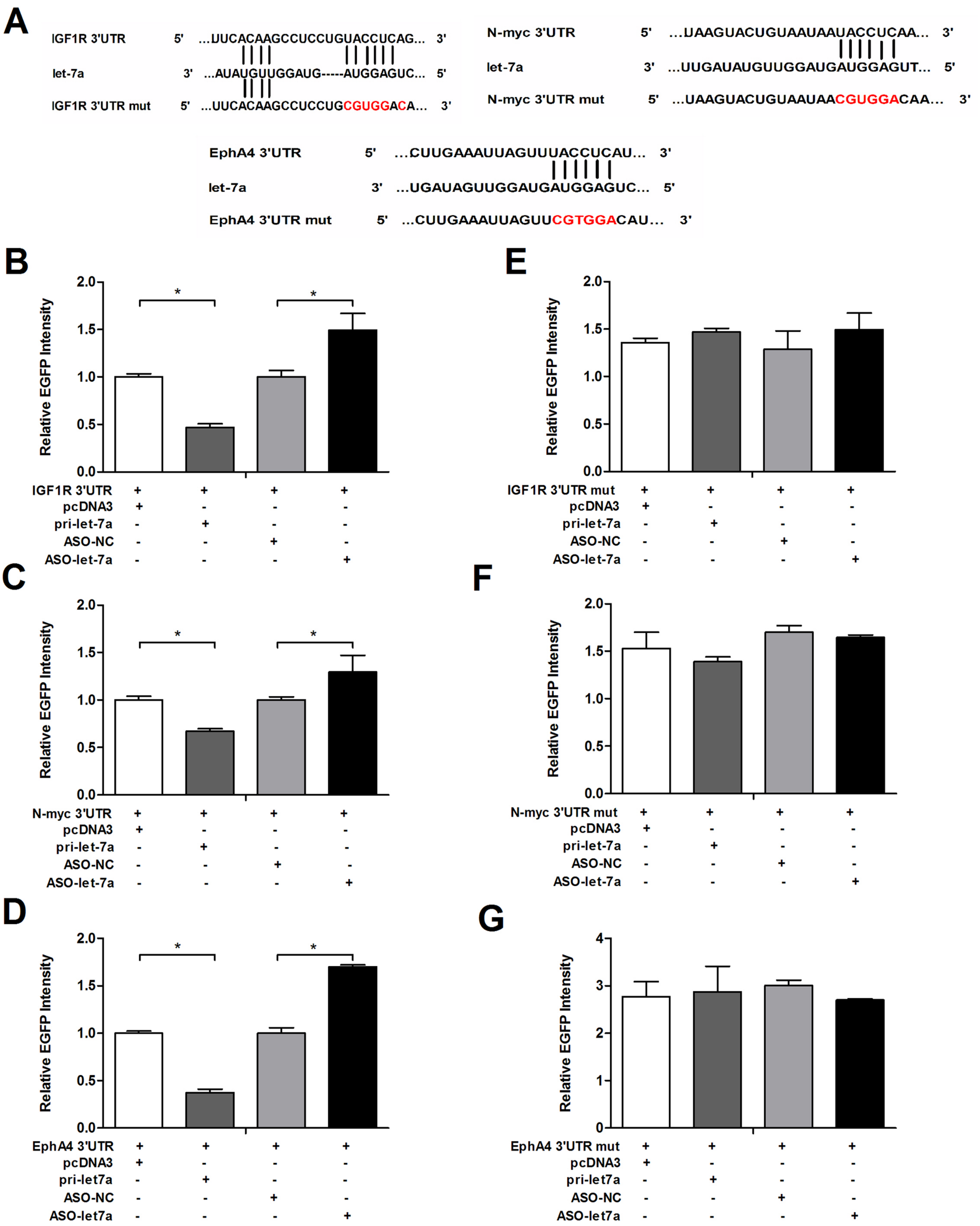

Supplementary Figure S4: Target genes of let-7a are confirmed in an EGFP reporter assay. A. The 3′-UTR and 3′UTR-mut of IGF1R, N-myc and EphA4 were cloned downstream of EGFP. **B-G.** These constructs were cotransfected into HeLa cells with plasmids overexpressing or blocking RSU1P2. An RFP plasmid was used as control. The intensities of EGFP were measured 48 h after transfection.

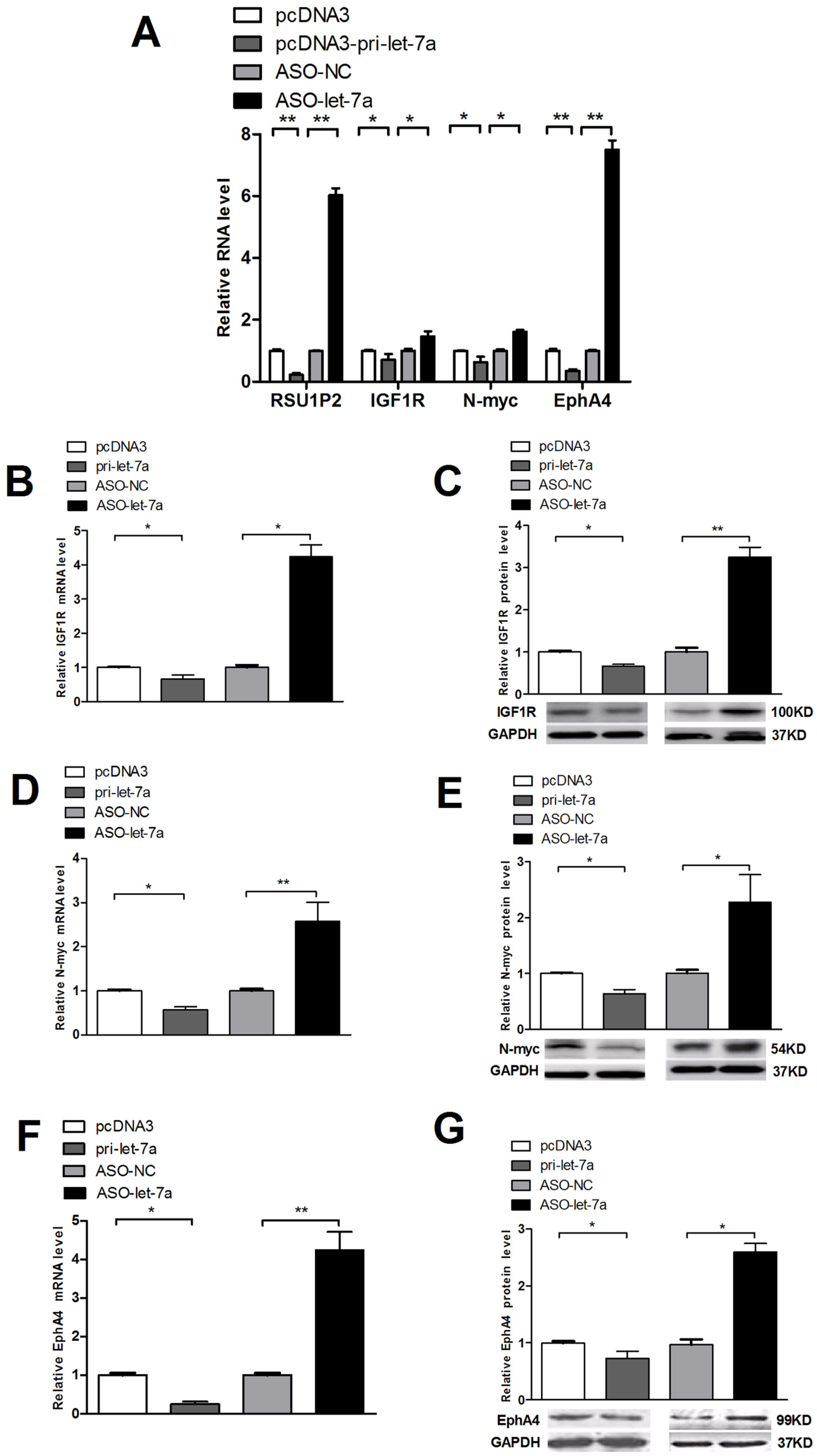

Supplementary Figure S5: let-7a downregulates endogenous mRNA and protein expression of its target genes. (S5A) qRT-PCR analysis showed the mRNA levels of RUS1P2, IGF1R, N-myc and EphA4 after alteration of let-7a levels. qRT-PCR and western blot showed mRNA and protein levels of the let-7a target genes, IGF1R (S5B, C), N-myc (S5D, E) and EphA4 (S5E, F) in HeLa cells transfected with pir-let-7a or ASO-let-7a. β-Actin was used as an internal control for normalization.

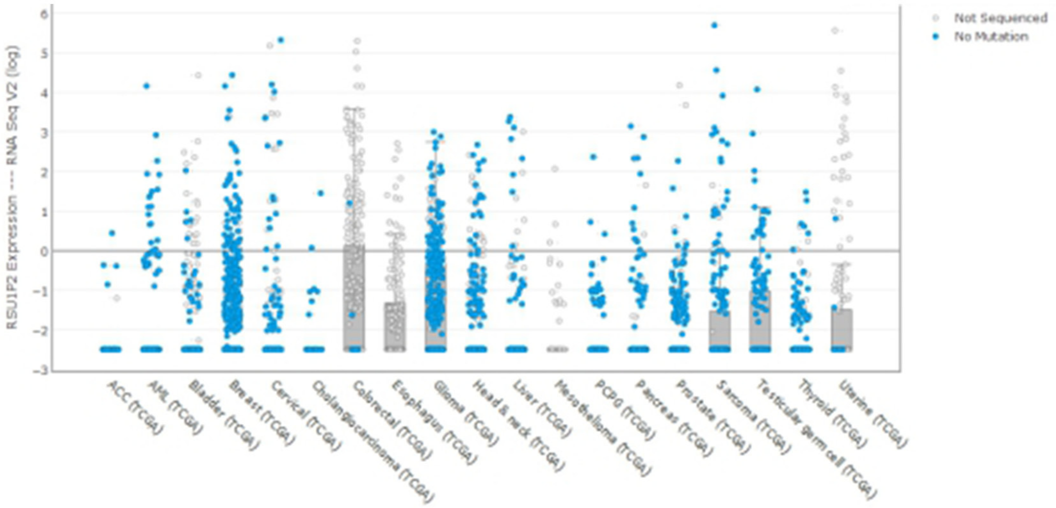

Supplementary Figure S6: The expression of RSU1P2 in serival cancers in. RSU1P2 is high-expression in breast cancer, bladder cancer, prostate cancer and other cancers.

Supplementary Table S1: The Primers and oligonucleotides used in this work.

See Supplementary File 1
