# Supplementary material for: LncRNA RSU1P2 contributes to tumorigenesis by acting as a ceRNA against let-7a in cervical cancer cells
Source: Oncotarget. 2016 Jul 26;8(27):43768–81. doi: 10.18632/oncotarget.10844 (PMC5546439; doi:10.18632/oncotarget.10844)
Supplement: Supplementary file 2 [file oncotarget-08-43768-s002.doc]

Table 1 The Primers and oligonucleotides used in this work

| Name | sequence |
| --- | --- |
| RUS1P2-qPCR-S | 5'-GGACATCGAGAAACTAAAG-3' |
| RUS1P2-qPCR-AS | 5'-GGTCACAGAACAAGGGAG-3' |
| β-actin-S | 5'-CGTGACATTAAGGAGAAGCTG-3' |
| β-actin-AS | 5'-CTAGAAGCATTTGCGGTGGAC-3' |
| pri-let-7a-S | 5'-GGAGAATTCGAAACCAGGATTACCGAGG-3' |
| pri-let-7a-AS | 5'-GGAGACTCGAGCAAATGCTGCACTACATCTC-3' |
| let-7a RT primer | 5'-GTCGTATCCAGTGCAGGGTCCGAGGTATTCGCACTGGATACGACAACTAT-3' |
| U6 RT primer | 5'-GTCGTATCCAGTGCAGGGTCCGAGGTATTCGCACTGGATACGACAAAATATGGAAC-3' |
| let-7a Forward primer | 5'-TGAGGTAGTAGGTTGT-3' |
| U6 Forward primer | 5'-TGCGGGTGCTCGCTTCGGCAGC-3' |
| U6 Reverse primer | 5′-CCAGTGCAGGGTCCGAGGT-3′ |
| Oligo dT primer | 5'-TTTTTTTTTTTTTTTT-3' |
| ASO-NC | 5′-UGACUGUACUGAGACUCGACUG-3 |
| ASO-let-7a | 5'-AACUAUACAACCUACUACCUCA-3’ |
| RSU1P2-let-7a-Top | 5'-GATCCGGCTCACTGCAACCTCTGCCTCCCAAGCTTG- 3' |
| RSU1P2-let-7a-Bot | 5'-AATTCAAGCTTGGGAGGCAGAGGTTGCAGTGAGCCG- 3' |
| RSU1P2-let-7a-mut-Top | 5’-GATCCGGCTCACTGTAGATTCTGATTCCCAAGCTTG- 3’ |
| RSU1P2-let-7a-mut-Bot | 5’-AATTCAAGCTTGGGAATCAGA ATCTACAGTGAGCCG- 3’ |
| RUS1P2-full-S | 5'CGGGATCCACTTGAATAACCAAACCTATGCC3' |
| RUS1P2-full-AS | 5'CCGGAATTCGAGAATTACTATGTGATTTGTACC3' |
| shR-RSU1P2-Top | 5'-GATCCGCAGTAGAGAATAGAGGTTACTCGAGTAACCTCTATTCTCTACTGTTTTTTG-3' |
| shR-RSU1P2-Bot | 5'-AATTCAAAAAACAGTAGAGAATAGAGGTTACTCGAGTAACCTCTATTCTCTACTGCG-3' |
| N-myc-3'UTR-Top | 5'-AATTC TAAGTACTGTAATAATACCTCAAAGCTTC-3' |
| N-myc-3'UTR-Bot | 5'-TCGAGAAGCT TTGAGGTATTATTACAGTACTTAG-3' |
| N-myc-3'UTR-mut-Top | 5'-AATTCTAAGTACTGTAATAACGTGGACAAGCTTC-3' |
| N-myc-3'UTR-mut-Bot | 5'-TCGAGAAGCTTGTCCACGTTATTACAGTACTTAG-3' |
| IGF1R-3'UTR-Top | 5'-AATTCCATTCACAAGCCTCCTGTACCTCAGAAGCTTC-3' |
| IGF1R-3'UTR-Bot | 5'-TCGAGAAGCTTCTGAGGTACAGGAGGCTTGTGAATGG-3' |
| IGF1R-3'UTR-mut-Top | 5'-AATTCCATTCACAAGCCTCCTGCGTGGACAAGCTTC-3' |
| IGF1R-3'UTR-mut-Bot | 5'-TCGAGAAGCTTGTCCACGCAGGAGGCTTGTGAATGG-3' |
| EphA4-3'UTR-Top | 5'-AATTCTCTTGAAATTAGTTTACCTCATAAGCTTC-3' |
| EphA4-3'UTR-Bot | 5'-TCGAGAAGCTTATGAGGTAAACTAATTTCAAGAG-3' |
| EphA4-3'UTR-mut-Top | 5'-AATTCTCTTGAAATTAGTTCGTGGACTAAGCTTC-3' |
| EphA4-3'UTR-mut-Bot | 5'-TCGAGAAGCTT AGTCCACGAACTAATTTCAAGAG-3' |
| N-myc-qCPR-S | 5'-TGAACACGCTCGGACTTG-3' |
| N-myc-qCPR-AS | 5'-TCCCAACCGTCACCAAC-3' |
| IGF1R-qCPR-S | 5'-CTTGATTGGTCTGGCTG-3' |
| IGF1R-qCPR-AS | 5'-GTGTGCCCCATAGCTGTC-3' |
| EphA4-qCPR-S | 5'-CAGAGGTAAGGGTAGGAGGC-3' |
| EphA4-qCPR-AS | 5'-AGCAGTGTAGCGAGCACAAC-3' |
| N-myc-EcoRI-S | 5'-GGAGAATTCATGCCGAGCTGCTCCACGTCCAC-3' |
| N-myc-XbaI-AS | 5'-GAGAATCTAGACGGCAAGTCCGAGCGTGTTCAATT-3' |
| shR-N-myc-Top | 5'-GATCCGCTGTTGAAGTCACCTTGTGTGTCTCGAGACACACAAGGTGACTTCAACAGCTTTTTGA-3' |
| shR-N-myc-Bot | 5'-AGCTTCAAAAAGCTGTTGAAGTCACCTTGTGTGTCTCGAGACACACAAGGTGACTTCAACAGCG-3' |
| EphA4-EcoRI-S | 5’-CCTCGCTCGAGGAATTCACCATGGCTGGGATTTTCTATTTC-3’ |
| EphA4-XhoI-AS | 5’-CCAGACTCGAGGCGACGGGAACCATTCTGCCGTG-3’ |
| shR-EphA4-Top | 5’-gatccGAGCGTTTCATCAGAGAGAttcaagagatctctctgatgaaacgctcTTTTTTGAATTC-3’ |
| shR-EphA4-Bot | 5’-TCGAGAATTCAAAAAAGAGCGTTTCATCAGAGAGAtctcttgaatctctctgatgaaacgctcg-3’ |
| RSU1P2-p2937-S | 5'-GGGGTACCCCAGCCCAGACAACACTGTGA-3' |
| RSU1P2-p2937-AS | 5'-GAAGATCTGTAGCAGTGCTTAGCTTCTTGCC-3' |
| RSU1P2-p2565-S | 5'-CGGGGTACCCATCTTAGATGTTCCTTT-3' |
